# Efficacy of Web-Based Supportive Interventions in Quality of Life in COPD Patients, a Systematic Review and Meta-Analysis

**DOI:** 10.3390/ijerph182312692

**Published:** 2021-12-02

**Authors:** Andrés Calvache-Mateo, Laura López-López, Alejandro Heredia-Ciuró, Javier Martín-Núñez, Janet Rodríguez-Torres, Araceli Ortiz-Rubio, Marie Carmen Valenza

**Affiliations:** Department of Physical Therapy, Faculty of Health Sciences, University of Granada, 18007 Granada, Spain; andrescalvache@ugr.es (A.C.-M.); lauralopez@ugr.es (L.L.-L.); ahc@ugr.es (A.H.-C.); javimartinn29@gmail.com (J.M.-N.); jeanette92@ugr.es (J.R.-T.); aortiz@ugr.es (A.O.-R.)

**Keywords:** communication, COPD patients, educational content, supportive interventions, web-based

## Abstract

Background: Adults living with Chronic Obstructive Pulmonary Disease (COPD) often have difficulties when trying to access health care services. Interactive communication technologies are a valuable tool to enable patients to access supportive interventions to cope with their disease. The aim of this revision and meta-analysis is to analyze the content and efficacy of web-based supportive interventions in quality of life in COPD. Methods: Medline (via PubMed), Web of Science, and Scopus were the databases used to select the studies for this systematic review. A screening, analysis, and assessment of the methodological quality was carried out by two independent researchers. A meta-analysis of the extracted data was performed. Results: A total of 9 of the 3089 studies reviewed met the inclusion criteria. Most repeated web content elements were educational and involved communication with healthcare professional content. Finally, seven of the nine studies were included in a quantitative analysis. Web-based supportive interventions significantly improved quality of life when added to usual care (SMD = −1.26, 95% CI = −1.65, −0.86; *p* < 0.001) but no significant differences were found when compared with an autonomous pedometer walking intervention (*p* = 0.64) or a face-to-face treatment (*p* = 0.82). Conclusion: This systematic review and meta-analysis suggests that web-based supportive interventions may complement or accompany treatments in COPD patients due to the advantages of online interventions. The results obtained should be treated with caution due to the limited number of studies in this area and methodological weaknesses.

## 1. Introduction

Chronic obstructive pulmonary disease (COPD) is a non-reversible inflammatory disease that causes progressive obstruction of the airways. According to the Global Initiative for Chronic Obstructive Lung Disease (GOLD) 2020 report, COPD is the leading lung disease in terms of mortality and morbidity worldwide [1,2]. Due to the increase in smoking and the progressive ageing of the population, the prevalence of COPD will increase in the coming years [3].

As the disease progresses, the symptoms become increasingly severe and complex. Often, the combination of psychological, emotional, and social factors with physical symptoms makes it difficult for patients and professionals to deal with the disease [4]. As a result, COPD patients experience significant impairment of disease-related quality of life as well as social isolation [5] that generates a significant burden of disability [6] and demands continuous health care [7].

Unfortunately, COPD patients face significant barriers when seeking access to appropriate health services to manage the disease, including living in medically underserved regions [8], language barriers [9], reduced mobility due to the disease itself, or other comorbidities, such as ageing and limited time [10]. In addition, due to the respiratory status of these patients and the potentially serious medical consequences for them, the risk of COVID-19 infection should be minimized [11,12]. Despite all these obstacles, there are not many interventions to support COPD patients in dealing with their disease [8].

Technological development is a great opportunity to generate new tools to support COPD patients [8,13]. Those technologies have enabled existing therapies to be delivered online and allow for the development of new interventions tailored to patients’ needs [14]. New technologies are increasingly being investigated with the aim of developing interventions that can adequately complement or replace interventions already provided in health services [15,16,17].

Rapid advances towards a more digitalized society as well as the rapid development of today’s electronic devices have caused a significant rise in the availability of communication technologies applied to health services [18,19]. The different online health communication tools allow patients to access personalized content, disease self-management tools, and communication with healthcare professionals from the comfort and security of their own home [20,21,22].

The most recent systematic reviews and meta-analyses [23,24,25,26,27,28,29,30,31,32,33] on telehealth care analyze teleassessment, telephone assistance, mobile app development, and website assistance in depth, but they need to be analyzed separately [8].

Previous studies show chronic disease patients’ need for personalized web-based interventions [34,35]. COPD patients demand access to information about their health status, related to the disease itself and to the improvement of quality of life [8]. Different mechanisms related to a perception of health-related needs, such as health education, self-management [36], and family and social support, have a significant influence on the quality of life of patients using web-based interventions [12,21,27,37,38,39].

Web-based interventions can encompass several distinct, often overlapping interventions, including: (1) tele-education content; (2) symptom and mood telemonitoring; (3) physical activity monitoring and personalized feedback to the patient; (4) tele-education in self-management skills; (5) tele-consultation with healthcare professionals; (6) tele-communication with other patients; (7) remote decision support systems; (8) tele-diagnosis; and (9) tele-rehabilitation [27,40,41].

The advantages offered by web-based interventions such as easy and on-demand access to health information content, interactive support with other patients, and tools for symptom self-management may have the potential to influence the different variables and symptoms of a COPD patient. There is a need to investigate whether these web-based interventions have an impact on the quality of life of COPD patients and determine which are the most appropriate contents. The aim of this revision and meta-analysis is to analyze the content and efficacy of web-based supportive interventions in quality of life in COPD.

## 2. Materials and Methods

### 2.1. Search Strategy and Eligibility Criteria

This systematic review was conducted according to the Preferred Reporting Items for Systematic Review and Meta-Analysis (PRISMA) statement guidelines [42] and its registration number in the International Prospective Register of Systematic Review (PROSPERO) is CRD42020211978. The Cochrane Collaboration guidelines for reviewing interventions were also closely followed [43]. Three databases were used for the electronic search: Medline (via Pubmed), Web of Science, and Scopus. The screening and analysis of the studies was conducted between November 2020 and March 2021. Relevant publications from inception to 1 March 2021 were included. A search strategy was created for Medline and then modified to be specific to each of the databases. The following Medical Subject Headings (MeSH) terms were used (Appendix A).

To adequately define the research question, the impact of patient, intervention, comparison, outcome (PICOS) strategy [44] was applied.

(P) Population: COPD patients over 18 years of age.

(I) Interventions: Studies that used web-based supportive interventions.

(C) Comparison: Non-web-based interventions.

(O) Outcome: Any outcome reporting quality of life (e.g., St. George Respiratory Questionnaire, Chronic Respiratory Disease Questionnaire).

(T) Timing: At any time.

(S) Setting: No restriction of setting.

Only full-text randomized controlled trials written in English, Spanish, and French were included in the systematic review. Systematic reviews and meta-analyses, observational studies, clinical practice guidelines, letters, abstracts, editorials, conference papers, theses, and dissertations were excluded. Studies in other languages were also considered for inclusion when translation was possible.

### 2.2. Study Selection and Data Extraction

After all studies had been retrieved from the three databases, duplicates were re-moved. To determine if the articles met the inclusion criteria for this systematic review, two independent investigators performed a first assessment of the title and abstract of all studies. If the article met the inclusion criteria, it was selected for a second phase in which the full text was analyzed and reviewed.

The Cochrane guidelines for systematic reviews were followed for data extraction [43]. A third reviewer was responsible for resolving any disagreement between the two main reviewers. The information extracted from the articles was: year of publication, main author, sample size, sample age, treatment status, severity of COPD, specific intervention for the control and experimental groups, web content elements, intervention duration, outcome measures, and main results. If the reviewers did not find any data during the analysis and review of the articles, they contacted the authors of the studies.

### 2.3. Assessment of Methodological Quality and Risk of Bias

The Downs and Black quality checklist was used to assess the methodological quality of the studies included in the review [45]. This assessment was carried out independently by the two principal investigators. This method contains 27 items divided into 5 subscales (study quality, external validity, study bias, confounding and selection bias, and study power). Due to its high reliability and validity, this scale is considered one of the six most appropriate scales to measure the quality of the studies included in a systematic review [46]. Studies are classified into four categories according to the score obtained: it will be classified as poor if its score is less than or equal to 14, fair if the score is between 15 and 19, good if the score is between 20 and 25, and excellent if the score is between 26 and 28 [46,47].

In addition to the methodological quality of the articles, the risk of bias was assessed using the Cochrane Risk of Bias Tool for Randomized Controlled Trials [43]. This measurement tool is divided into seven items that are subdivided into six subscales. The first subscale corresponds to the selection bias and is the only one with two items. The remaining subscales are called performance bias, detection bias, attrition bias, reporting bias, and other bias, and have only one item. When the reviewer determines that there is a low risk of bias for each of the items, the study is classified as high quality. When the reviewer determines that one of the items is not met because there is a high risk of bias or two of the items cannot be answered clearly, the study is classified as fair quality. When the reviewer determines that one of the items is not met because there is a high risk of bias or two of the items cannot be answered clearly and there are important limitations that may invalidate the results, the study is classified as poor quality. The study is also classified as poor quality when two or more items are not met.

### 2.4. Data Synthesis and Analysis

A meta-analysis was undertaken using Review Manager (RevMan v5.3; Cochrane Collaboration, Oxford, UK). All variables included were continuous data. Study authors were contacted by e-mail whenever data were insufficient for the purposes of meta-analysis (e.g., neither means nor standard deviation were provided). Authors were given 2 weeks to respond. If they had not responded within a week, they were written to again as a reminder. The embedded Review Manager calculator was used to calculate standard deviations whenever *p*-values or 95% confidence intervals were given [48].

The main outcome considered for this meta-analysis was quality of life. Standardized mean differences were used because all scales were assumed to measure the same underlying symptom or condition, but some studies measured outcomes on different scales and 95% confidence intervals (CI) were calculated for all outcomes [49]. Subgroup analysis was also used in this study to help clarify the different uses of web-based interventions.

When the studies presented different scales to measure quality of life, we selected the data provided by the Saint George Respiratory Questionnaire (SGRQ), since it is the most frequently used, disease-specific quality of life measure in this population group [50]. When studies did not use the SGRQ, scores from other disease-specific quality of life scales, such as the Chronic Respiratory Disease Questionnaire (CRQ), were used [50,51,52]. The scoring of the different scales was converted so that a lower score always indicated a better outcome.

The Q and I^2^ statistics were calculated to examine statistical heterogeneity, and a visual inspection of the forest plots was also performed to identify outlier studies. The I^2^ is a statistical value that is interpreted as the percentage of the total variation observed between studies that is due to the difference between them and not to sampling error (chance). An I^2^ of ≥50%; I^2^ >25% and < 50%; I^2^ of ≤25% were considered to indicate high, moderate, and low heterogeneity, respectively. When the I^2^ value is greater than 50%, the meta-analysis is considered heterogeneous and, therefore, a random effects analysis had to be used. Statistical significance was established as *p* < 0.05, which means that the effects differ significantly between the control and intervention groups. We also explored sources of heterogeneity and performed a sensitivity analysis excluding trials with high risk of attrition or detection bias [48].

## 3. Results

### 3.1. Study Selection

**Figure 1:**
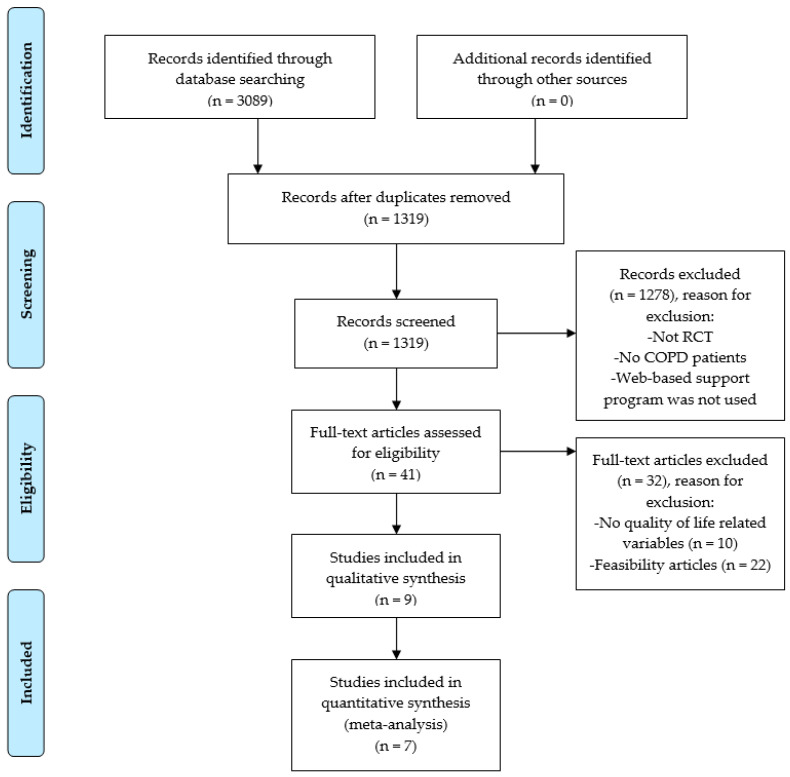
PRISMA flow chart of the literature screening process and results.

An initial search of the databases found 3089 records. After eliminating duplicates, a total of 1319 studies were selected. In the end, an overall total of 9 studies that analyzed a total of 1168 participants were included in this systematic review. The PRISMA flow diagram for the study selection process is shown in Figure 1.

### 3.2. Study Characteristics

Table 1 shows the main characteristics of the studies included in the systematic review. The included studies were published between 2013 and 2020, and assessed participants were aged between 66.1 [53] and 71.9 years [54]. All the studies except the study of Wang et al. (47.5%) [54] had a higher proportion of males than females in the study sample. Regarding COPD severity, five studies [55,56,57,58,59] included mild to very severe patients and four studies [53,54,60,61] included moderate to very severe patients. All studies included clinically stable patients, with the exception of Wang et al. [54] and Jiménez-Reguera et al. [61], which included patients after discharge.

The web-based supportive interventions of each study were covered in Table 2 by the content of the comparison group approach, the content of the experimental interventions, the intervention duration, the outcome measures, and main results. Table 2 also includes nine web content elements that were identified as important to the technical characteristics of internet-supported therapeutic interventions [27,62] as well as for evidence-based web interventions: 1, tele-education content; 2, symptom and mood telemonitoring; 3, physical activity monitoring and personalized feedback to the patient; 4, tele-education in self-management skills; 5, tele-consultation with healthcare professionals; 6, tele-communication with other patients; 7, remote decision support systems; 8, tele-diagnosis and 9, tele-rehabilitation [63,64].

One study compared the usual care with a comparator group who received the usual care in addition to the web-based supportive program based on tele-education and tele-consultation with healthcare professionals [54]. Four studies compared a web-based supportive pedometer walking intervention based on physical activity monitoring, personalized feedback to the patient, and tele-education, with a pedometer walking intervention without web support [56,57,58,59].

Four studies attempted to demonstrate the non-inferiority of the web-based intervention when compared to a face-to-face program. For this purpose, the same intervention was carried out in both face-to-face and online modalities. Two studies were based on a telerehabilitation program [53,60], another in a self-management program [55], and the last one was based on tele-education and symptom and mood telemonitoring [61].

Most repeated web content elements were tele-education content, self-management skills training, and tele-consultation with healthcare professionals. Only one study [57] excluded educational content. Education in self-management skills and tele-communication with healthcare professionals were excluded by Jiménez-Reguera et al. [61]. in three of the studies [53,55,60].

In each study, the mean duration of intervention was 7.9 months (ranging from 6 weeks to 15 months). Most of the studies conducted an intervention over one year [54,55,57,59]. One study conducted an intervention of 10 months [61] and 4 studies conducted an intervention of less than 4 months [53,56,58,60].

The included studies evaluated quality of life using different tools. Disease-specific tools, e.g., the St. George’s Respiratory Questionnaire (SGRQ), Chronic Respiratory Questionnaire (CRQ), Chronic Obstructive Pulmonary Disease Assessment Test (CAT) and general tools, e.g., the Short Form 36-Item Health Survey (SF-36) and EuroQol 5-Dimension Questionnaire (EQ- 5D) were used. The most commonly reported outcome was SGRQ, which was followed by CRQ and CAT.

Other variables used in several studies were: self-efficacy, functional capacity, dyspnea, physical activity, lung function, anxiety, and depression. Self-efficacy was measured in four studies, with the most used tool being the Exercise Self-Regulatory Efficacy Scale (Ex-SRES). Functional capacity was the second most frequently measured variable after quality of life. Five studies measured functional capacity with the 6MWT being the most used tool [54,55,58,60,61]. Four studies measured dyspnea and physical activity [54,55,58,60], three studies measured anxiety and depression [53,58,60] and two studies measured lung function [54,61].

The results obtained in the majority of included RCTs show no significant differences between groups in quality of life. Only one study reaches significant results in quality of life when compared to control intervention [54]. This result can be due to the duration of the program (12 months) and the content related to coaching. Furthermore, the majority of included studies showed significant improvements among the group in quality of life outcomes after intervention [53,55,56,57,61]. In addition, some studies aimed to demonstrate that web-based intervention was not inferior to face-to-face intervention and found similar results in quality of life for the intervention and control groups [53,55,60,61].

Regarding the results of other outcomes, most of the included studies in this systematic review have significant results in a functional capacity. Four studies [54,56,57,58] were significant between group results in favor of the web-based intervention group and three studies were significant among group improvements in a functional capacity after intervention for the web-based group [53,55,61]. Studies intended to demonstrate the non-inferiority of web-based support intervention found similar functional capacity results for the intervention and control groups.

Nguyen et al. [55] showed a significant improvement in dyspnea compared with the baseline in the experimental group and Wang et al. [54] showed a significance between the group’s difference in dyspnea and lung function in favor of the experimental group.

### 3.3. Risk of Bias

**Figure 2:**
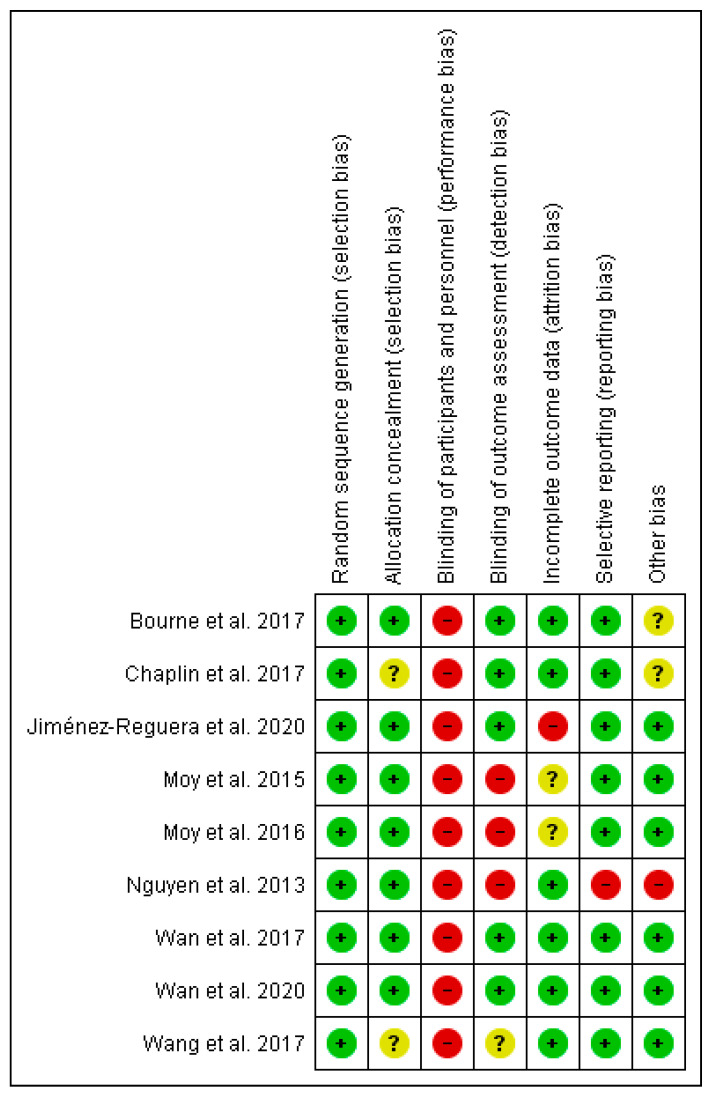
Risk of bias assessment of included studies. Notes: red, high risk of bias; yellow, moderate risk of bias; green, low risk of bias.

The Downs and Blacks scale scores are presented in Table 1. The average score of the included studies in this systematic review was 21.6 points. In accordance with the suggested cut-off points to grade studies according to methodological quality, one article was rated as “fair” (15–19 points) [61] and eight were categorized as “good” (20–25 points) [53,54,55,56,57,58,59,60]. Figure 2 shows, in detail, the scoring of the studies on the different items of the Cochrane Risk of Bias Tool for randomized trials.

### 3.4. Results of Meta-Analysis

Data from seven RCTs reporting results obtained in quality of life were included in the meta-analysis [54,55,56,57,58,60,61]. All the included studies use the SGRQ to measure quality of life, except for the study conducted by Nguyen et al. [55] which used the CRQ.

All studies that did not provide sufficient data on quality of life (means and standard deviations at baseline or after the intervention) and for which no response was received from the authors were excluded. Ultimately, the analysis has been performed on a total of 873 patients (359 for control and 514 for intervention).

**Figure 3:**
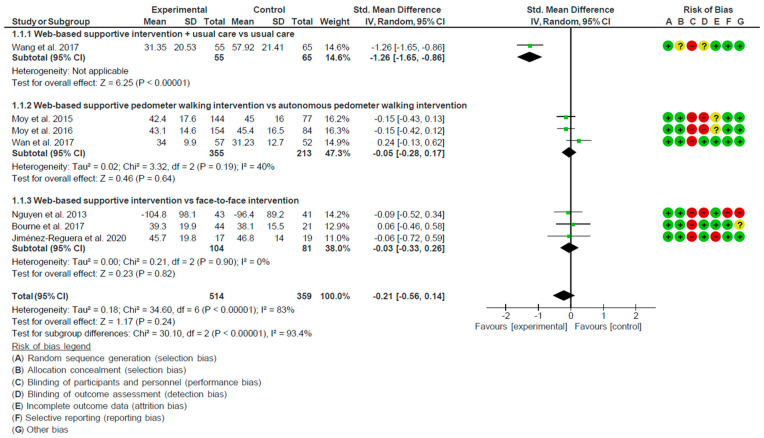
Forest plot of the effect of web-based supportive interventions on quality of life in COPD patients compared with the control group. Notes: Risk of bias color: red, high risk of bias; yellow, moderate risk of bias; green, low risk of bias.

Figure 3 depicts the forest plot. Due to the statistical heterogeneity of the results (I^2^ = 83%, *p* < 0.001), a statistical random effects model was applied. Patient quality of life was not significantly improved in the intervention groups in comparison with controls (SMD = −0.21, 95% CI = −0.56, 0.14).

When compared to usual care, the mean difference showed a significant overall effect with the addition of the web-based supportive program to usual care (SMD = −1.26, 95% CI = −1.65, −0.86; *p* < 0.001, one study [54]). When compared to a pedometer walking intervention without web-support with a web-based supportive pedometer walking intervention (SMD = −0.05, 95% CI = −0.28, 0.17; *p* = 0.64, three studies [56,57,58]) or a web-based supportive intervention with a face-to-face intervention (SMD = −0.03; 95% CI= −0.33, 0.26; *p* = 0.82, three studies [55,60,61]), the pooled SMD showed no significant overall effect.

## 4. Discussion

The continuous technological growth of today’s society, the increasing use of online services, and patients’ need for new supportive solutions have facilitated the creation of new web-based interventions that have not been properly tested yet. To the authors’ knowledge, this is the first systematic review and meta-analysis evaluating the effects of web-based supportive interventions on quality of life in COPD patients.

Our results support the idea that web-based supportive interventions can improve the quality of life in COPD patients. Nevertheless, it is important to note that the systematic review of the literature related to the design of web-based supportive interventions must be correctly interpreted, considering the different sample sizes of the studies, the differences in length of therapy and follow up, and the differences in effect size of the included studies.

Our systematic review is the first one specifically exploring the effects of web-based supportive interventions in quality of life in COPD patients, with nine RCTs [53,54,55,56,57,58,59,60,61] included in the qualitative analysis. Our results are consistent with those of previous systematic reviews performed in COPD patients and other telehealth systems [23,34,65,66,67,68].

Internet-based interventions can, however, present a rather confusing picture as the only common ground is the delivery medium. The interventions may range from posting pamphlets online to dynamic combinations of text-based information and communicative features, such as forums, “ask an expert”, or multimedia tools, to individually computer tailored content [69].

Regarding web components, Sobnath et al. [70] described the possible features that a potential supporting tool for COPD patients should have in their systematic review. The tools must be easily accessible both for patients and health professionals. In addition, they should be adapted to elderly patients with limited experience in the use of technology and have a user-friendly interface. According to previous literature, the tool should include a customized education section for each patient, with disease-specific information and self-management material, phycological motivation to encourage good adherence, electronic coaching, comment sections, and social networks to share information with health professionals [70,71].

Among the web-based supportive interventions analyzed, the educational content was the most used alone or in combination with other contents, and the most frequent comparison treatment was the same in a face-to-face format. When compared, web-based supportive interventions showed similar results in all measured variables.

The web-based support interventions analyzed in this systematic review used a variety of components of COPD patient support tools that were described by Sobnath et al. [70], such as personalized education sections and social networks to share information with medical professionals. Our results are in line with the previous systematic reviews conducted in patients with cancer in which the most common and promising interventions include a combination of effective communication with healthcare providers, customized educational strategies based on the patient’s disease and condition, ongoing symptom monitoring, disease self-management tools, and automated feedback [72,73].

It is difficult to determine exactly which web elements are most important in designing an effective disease management tool, and to determine whether the effects are due to one or some of the elements, or to all of them together. Effective communication with healthcare providers is highly recommended content for web-based support intervention since patients have different characteristics, preferences, and needs [62,74] as seen in the Norwegian WebChoice study [75].

A Cochrane review identified that in improving the quality of life of COPD patients, the effects of technology-based interventions attenuated over time. Support interventions based on new technologies were found to be more effective in improving the quality of life of COPD patients than interventions based on face-to-face education and support materials even at six months, but not at one year. This is probably due to the fact that educational and motivational content were not updated during the maintenance phase [67,76], highlighting the importance of these elements.

Our systematic revision of web-based interventions in COPD, have shown additional improvements in dyspnea and physical activity in programs which include self-management components [54,55,56,58,59]. Different reviews [13,17,19,77] have reported the opportunities for telehealth interventions in increasing physical activity and symptoms when behavioral components are included.

Given the great heterogeneity and diversity of the studies included in this systematic review, it might not be recommended to perform a meta-analysis. However, a random-effects model was chosen to allow the pooling of more clinically heterogeneous studies [78]. Furthermore, to adequately answer the question discussed in this review, i.e., whether web-based support interventions are effective in improving the quality of life of COPD patients, and due to the great diversity of the studies published to date, it was necessary to use a wide range of studies in which these types of interventions were used. It is therefore required to adequately justify our findings.

The findings of our meta-analysis of pooled data do not identify statistically significant differences in the quality of life of COPD patients. Even though the results of this meta-analysis suggested that there is no evidence that web-based support interventions are effective in increasing the quality of life of COPD patients, the results should be analyzed by subgroups.

This meta-analysis supports the promising role and the feasibility of web-based supportive interventions in COPD patients to improve quality of life when added to the usual care, reaching the currently minimum significant established difference for SGRQ results in a mean COPD sample population of −4 points [79], but not when compared to an autonomous pedometer walking intervention or face-to-face treatment. These results are in line with the increasing evidence in literature on the success of telehealth interventions [64,65,66,67].

Four included studies used wearable systems like the pedometer in the web-based supportive programs [56,57,58,59]. Those programs showed similar results in quality of life to those using autonomous interventions. Those results can be due to the theory of self-regulation [80], in which the use of a pedometer (either web-based or autonomous) guides the patient to their own feelings, thoughts, and behaviors to achieve specific goals. In addition, blinding patients from the web-based supportive pedometer walking interventions would require giving a pedometer to the control group; this may cause the results of the control group to be altered, since the simple fact of having control of their daily steps may promote an increase in the physical activity of the patients.

Other studies have used web-based pulmonary rehabilitation programs compared to the same program developed face-to-face. The results obtained by Bourne et al. [60] show no significant differences between groups in quality of life. In the study by Jiménez-Reguera et al. and Nguyen et al. [55,61], the results show statistically significant improvement on the quality of life of the web-based group, but no differences between groups after intervention.

These studies support the argument that comparable results between web-based and face-to-face interventions, or the absence of impairment can be considered a success as seen in previous reviews [23,81], due to the opportunities for new technologies for at risk COPD patients [23,82]. In this line of thinking, web-based supportive interventions may complement routine care as no significant differences were found between the face-to-face and online modalities [70]. Some further advantages should be derived from the use of telehealth interventions for this argument to be valid and the extensive literature on this topic leaves no doubt. Telehealth intervention groups show better results than the control group in risk of exacerbation [83], costs of health care [84], hospitalization days [83], risk of hospitalizations, and risks of the emergency department visit, without the need for travel [85].

Our results are consistent with the increasing evidence in the literature on the efficacy of telehealth supportive interventions [23,34,65,66,67]. The use of web-based supportive interventions for COPD patients is not recommended if based solely on quality of life data, but there is also no argument against the use of these interventions.

Regarding the methodological quality, the seven RCTs included in the meta-analysis [54,55,56,57,58,60,61] were classified as “poor quality” according to the Downs and Black quality checklist. The main reason for the low quality of the studies included in this systematic review lies in methodological issues. For example, it has been shown in previous studies that selection bias in interventions based on technological tools is evident. The reason for this is that some patients are already used to the use of new technologies and the Internet, leading to the automatic preference of these over other tools [86].

In addition, web-based interventions appear to be unsuitable for all patients because the level of follow-up and adherence to treatments is often low [87]. Other factors that also increase the risk of bias involve the lack of patient blinding and not adequately describing the randomization method.

### Strengths and Limitations

To start with, we need to assess the strengths of the present study. First, only RCTs were included to increase the quality of evidence, and second, we were able to pool data from seven studies in a meta-analysis.

Thirdly, in previous studies on the effects of e-health’s intervention, web-based supportive intervention was not separately analyzed. In this study, web-based supportive intervention was first taken as a primary intervention.

The major weakness of this systematic review is the limited number of RCTs focused on web-based supportive interventions. However, the inclusion criteria enabled us to include articles with this type of intervention even if quality of life was not the main variable. There are no obvious reasons for the lack of research on COPD web-based supportive interventions but the issue of possible facilitators, such as a decreased burden of web-based interventions and the personalized nature and possible barriers including security and technical issues, should be addressed when performing these types of health interventions [88].

Other limitations need to be reported. First, one subgroup in our meta-analysis only had one study. Second, it should be noted that the diversity of the targeted interventions makes it difficult to distinguish whether the web-based supportive intervention was solely responsible for the observed effects. Third, since the authors were only fluent in French, English, and Spanish, they were only able to review research published or translated into these languages and not studies in other languages.

## 5. Conclusions

This systematic review and meta-analysis show the promising potential of web-based supportive interventions for improving quality of life in COPD patients. Due to the methodological limitations, the heterogeneity, and the limited number of studies in this field, the results should be treated with caution. Further randomized controlled studies are needed to evaluate the effect of web-based supportive interventions, with larger COPD populations and using appropriate interventions to blind the control group, thus increasing the evidence in this field of research.

### Practical Implications

Our findings suggest that the most common and promising web-based supportive intervention content are the educational content as well as communication with healthcare professionals. This systematic review and meta-analysis suggest that web-based supportive interventions may complement or accompany treatments in COPD patients due to the advantages of online interventions.

## Figures and Tables

**Table 1:** Characteristics of the included studies.

Study (Year)	Sample Size, Distribution and Sample Age *n* (% Men): (Mean ± SD)	Treatment Status	Severity	Downs and Black (Risk of Bias)
Nguyen et al. (2013) [55]	125 (54%) allocated randomly into: EG: 68.5 ± 11.0 CG1: 68.2 ± 9.9 CG2: 69.3 ± 8.0	Clinically stable	Mild to very severe	22 (Poor quality)
Moy et al. (2015) [56]	238 (93.7%) allocated randomly into: EG: 67.0 ± 8.6 CG: 66.4 ± 9.2	Clinically stable	Mild to very severe	22 (Poor quality)
Moy et al. (2016) [57]	238 (93.7%) allocated randomly into: EG: 67.0 ± 8.6 CG: 66.4 ± 9.2	Clinically stable	Mild to very severe	23 (Poor quality)
Wang et al. (2017) [54]	120 (47.5%) allocated randomly into: EG: 69.3 ± 7.8 CG: 71.9 ± 8.1	After discharge	Moderate to very severe	20 (Fair quality)
Wan et al. (2017) [58]	109 (98,2%) allocated randomly into: EG: 68.4 ± 8.7 CG: 68.8 ± 7.9	Clinically stable	Mild to very severe	23 (Fair quality)
Bourne et al. (2017) [60]	90 (65.56%) allocated randomly into: EG: 69.1 ± 7.9 CG: 71.4 ± 8.6	Clinically stable	Moderate to very severe	22 (Fair quality)
Chaplin et al. (2017) [53]	103 (68.93%) allocated randomly into: EG: 66.4 ± 10.1 CG: 66.1 ± 8.1	Clinically stable	Moderate to very severe	22 (Fair quality)
Wan et al. (2020) [59]	109 (98.17%) allocated randomly into: EG: 68.4 ± 8.7 CG: 68.7 ± 7.9	Clinically stable	Mild to very severe	23 (Fair quality)
Jiménez-Reguera et al. (2020) [61]	36 (61.11%) allocated randomly into: EG: 68.1 ± 6.6 CG: 68.1 ± 7.0	After discharge	Moderate to very severe	18 (Poor quality)

Notes: EG: experimental group; CG: control group; SD: standard deviation.

**Table 2:** Characteristics of the included studies in this systematic review.

Study	Interventions	Web Content Elements									Experimental Intervention Content	Intervention Duration	Outcomes Measures	Main Results
Nguyen et al. (2013) [55]	EG: internet-based dyspnea self-management program. CG1: face-to-face dyspnea self-management program. CG2: general health education.	1	2	3	4	5	6	7		9	Intervention included a personalized education program, dyspnea self-management training, exacerbation guidelines, personalized exercise with biweekly feedback and support, personal symptom and exercise log, real-time follow up, convenient access to information and support materials.	12 months	Quality of life measure(s): CRQ, SF-36. Other outcomes (measure(s)): self-efficacy (validated question); functional capacity (6MWT, ITT); dyspnea with activities (CRQ-D); arm endurance; adherence; satisfaction.	Quality of life results: No significant differences were found between groups in quality of life. EG participants had significant improvement in quality of life compared with baseline. Other outcomes results: Self-efficacy for managing dyspnea improved for the EG and CG1 compared with CG2. No significant differences were found in dyspnea and functional capacity between groups. EG participants had significant improvement in dyspnea and functional capacity compared with the baseline.
Moy et al. (2015) [56]	EG: web-based pedometer walking intervention. CG: pedometer walking intervention.	1	2	3	4	5	6				Step counting allowed for patient self-monitoring, new personalized weekly objectives were established, educational and motivational content to improve patient self-management, social support through an online forum.	4 months	Quality of life measure(s): SGRQ. Other outcomes (measure(s)): physical activity (pedometer); adherence; safety.	Quality of life results: No significant differences were found between groups in SGRQ total score. EG had significant improvement on symptoms and impact subscales compared to the CG. EG participants had significant improvement in SGRQ total score, symptoms, and impact compared with the baseline. Other outcomes results: EG had significant improvement on physical activity compared to the CG.
Moy et al. (2016) [57]	EG: web-based pedometer walking intervention. CG: pedometer walking intervention.		2	3	4	5	6				Step counting allowed for patient self-monitoring, new personalized weekly objectives were established, motivational content to improve patient self-management, social support through an online forum.	12 months	Quality of life measure(s): SGRQ. Other outcomes (measure(s)): physical activity (pedometer); adherence; safety.	Quality of life results: No significant differences were found between groups in quality of life. EG participants had significant improvement in SGRQ total score, symptoms, and impact compared with the baseline. Other outcomes results: Significant differences were found in physical activity between groups at month 4, but not in months 8 and 12.
Wang et al. (2017) [54]	EG: web based coaching program + routine care CG: routine care	1			4	5					These were used to manage patients’ clinical and demographic variables and enabled communication between health care providers and patients. The patient was able to access disease information, pulmonary rehabilitation instructions, and particular management of the participant was determined according to the evolution of the disease.	12 months	Quality of life measure(s): SGRQ. Other outcomes (measure(s)): functional capacity (6MWT); dyspnea (MRC); lung function (spirometry).	Quality of life results: EG had significant improvement in the SGRQ total score, SGRQ symptoms, SGRQ activity and SGRQ impact compared to the CG. Other outcomes results: EG had significant improvement of lung function, functional capacity, and degree of dyspnea compared to CG.
Wan et al. (2017) [58]	EG: web-based pedometer walking intervention. CG: pedometer walking intervention.	1	2	3	4	5	6				Step counting allowed for patient self-monitoring, new personalized weekly objectives were established, educational and motivational content to improve patient self-management, social support through an online forum.	3 months	Quality of life measure(s): SGRQ. Other outcomes (measure(s)): self-efficacy (Ex-SRES); functional capacity (6MWT); physical activity (pedometer); dyspnea (MRC); depression (BDI-II); COPD knowledge (BCKQ); social support (MOS-SSS); motivation and confidence to exercise; adherence.	Quality of life results: No significant differences were found between groups in quality of life. Other outcomes results: EG had significant improvement of daily step count compared to CG. No significant differences were found between groups in functional capacity, self-efficacy, dyspnea, depression, COPD knowledge, social support motivation, and confidence to exercise.
Bourne et al. (2017) [60]	EG: online supportive pulmonary rehabilitation. CG: face-to-face- supportive pulmonary rehabilitation.	1		3	4	5				9	Intervention included pulmonary online rehabilitation and educational videos to promote self-management.	6 weeks	Quality of life measure(s): SGRQ, CAT. Other outcomes (measure(s)): functional capacity (6MWT); dyspnea (MRC); anxiety and depression (HADS); adherence; safety.	Quality of life results: No significant differences were found between groups in quality of life. Other outcomes results: No significant differences were found between groups in exercise capacity, anxiety, and depression.
Chaplin et al. (2017) [53]	EG: web based pulmonary rehabilitation program. CG: face-to-face pulmonary rehabilitation program.	1	2		4	5	6	7		9	Intervention included education content, exacerbation guidelines, a home exercise program and goal setting, record of the progress, motivational interviewing techniques, and convenient access to information and support.	6–8 weeks	Quality of life measure(s): CRQ, CAT, EQ-5D. Other outcomes (measure(s)): self-efficacy (PRAISE); exercise capacity (ISWT, ESWT); anxiety and depression (HADS); COPD Knowledge (BCKQ).	Quality of life results: No significant differences were found between groups in quality of life. EG and CG participants had significant improvement in quality of life compared with the baseline. Other outcomes results: No significant differences were found between groups in any other outcome. EG and CG participants had significant improvement in functional capacity compared with the baseline.
Wan et al. (2020) [59]	EG: web-based pedometer walking intervention. CG: pedometer walking intervention.	1	2	3	4	5	6				Step counting allowed for patient self-monitoring, new personalized weekly objectives were established, educational and motivational content provided to improve patient self-management, social support through an online forum.	15 months	Quality of life measure(s): SGRQ. Other outcomes (measure(s)): self-efficacy (Ex-SRES); physical activity (pedometer); acute exacerbations.	Quality of life results: No significant differences were found between groups in quality of life. CG participants had a significant worsening of quality of life compared with the baseline. There was no significant change in EG group, indicating no significant decline. Other outcomes results: No significant differences were found between groups in daily step count and self-efficacy. EG participants had significant improvement of acute exacerbations compared with baseline. EG participants had a minor decline that CG participants in daily step count compared with baseline.
Jiménez-Reguera et al. (2020) [61]	EG: web-based follow-up program. CG: face-to-face follow-up program.	1	2								Intervention included an educational program and data collection related to disease and physical activity, daily reminders of daily exercise, record of medication intake, daily mood, and level of tiredness.	10 months	Quality of life measure(s): SGRQ, CAT, EQ- 5D. Other outcomes (measure(s)): functional capacity (6MWT); lung function (spirometry); adherence (CAP FISIO); adherence to physical activity (Morisky–Green Test).	Quality of life results: No significant differences were found between groups in quality of life. EG participants had a significant improvement of quality of life in compared with the baseline. Other outcomes results: No significant differences between the two groups were observed in functional capacity and lung function. EG participants had significant improvement of functional capacity in compared with baseline. EG participants had a significant improvement of adherence to the program and adherence to physical activity in compared with CG.

Notes: 1, tele-education content; 2, symptom and mood telemonitoring; 3, physical activity monitoring and personalized feedback to the patient; 4, tele-education in self-management skills; 5, tele-consultation with healthcare professionals; 6, tele-communication with other patients; 7, remote decision support systems; 9, tele-rehabilitation; EG, Experimental Group; CG, Control Group; CRQ, Chronic Respiratory Questionnaire; SF-36, Short Form 36 survey tool version 1; 6MWT, 6-Minute Walk Test; ITT, Incremental Treadmill Test; CRQ-D, Chronic Respiratory Questionnaire Dyspnea subscale; SGRQ, St. George’s Respiratory Questionnaire; MRC, Medical Research Council scale; Ex-SRES, Exercise Self-Regulatory Efficacy Scale; BDI-II, Beck Depression Inventory-II; BCKQ, Bristol COPD Knowledge Questionnaire; MOS-SSS, Medical Outcomes Study Social Support Survey; CAT, COPD Assessment Test; HADS, Hospital Anxiety and Depression Scale; EQ- 5D, EuroQol 5-Dimension questionnaire; PRAISE, PR Adapted Index of Self-Efficacy; ISWT, Incremental Shuttle Walk Test; ESWT, Endurance Shuttle Walk Test; CAP FISIO, Respiratory Physiotherapy Adherence self-report questionnaire.

## Appendix A

(“Chronic Obstructive Lung Disease” OR Airflow Obstruction, Chronic” OR “Chronic Airflow Obstructions” OR “Chronic Airflow Obstruction” OR “COPD” OR “Chronic Obstructive Pulmonary Disease” OR “COAD” OR “Chronic Obstructive Airway Disease” OR “Airflow Obstructions, Chronic”) AND (“eHealth” OR “ehealth” OR “e-Health” OR “e-health” OR “telemedicine” OR “tele-medicine” OR “Mobile Health” OR “Health, Mobile” OR “mHealth” OR “m-Health” OR “m-health” OR “telehealth” OR “tele-health” OR “telecare” OR “tele-care” OR “telemonitoring” OR “tele-monitoring” OR “teleconsultation” OR “tele-consultation” OR “health informatics” OR “internet” OR “mobile”) AND (“Life Quality” OR “Health-Related Quality Of Life” OR “Health Related Quality Of Life” OR “HRQOL” OR “quality of life” OR “management” OR “adherence” OR “healthy lifestyle” OR “well-being”).

## References

[1] Global Initiative for Chronic Obstructive Lung Disease GOLD Report 2020. https://goldcopd.org/wp-content/uploads/2019/12/GOLD-2020-FINAL-ver1.2-03Dec19_WMV.pdf.

[2] Gunasekaran K., Ahmad M., Rehman S., Thilagar B., Gopalratnam K., Ramalingam S., Paramasivam V., Arora A., Chandran A. (2020). Impact of a Positive Viral Polymerase Chain Reaction on Outcomes of Chronic Obstructive Pulmonary Disease (COPD) Exacerbations. Int. J. Environ. Res. Public Health.

[3] Lopez A.D., Shibuya K., Rao C., Mathers C.D., Hansell A., Held L.S., Schmid V., Buist S. (2006). Chronic obstructive pulmonary disease: Current burden and future projections. Eur. Respir. J..

[4] Disler R.T., Gallagher R.D., Davidson P.M. (2012). Factors influencing self-management in chronic obstructive pulmonary disease: An integrative review. Int. J. Nurs. Stud..

[5] Ghobadi H., Ahari S.S., Kameli A., Lari S.M. (2012). The Relationship between COPD Assessment Test (CAT) Scores and Severity of Airflow Obstruction in Stable COPD Patients. Tanaffos.

[6] Izquierdo J.L., Morena D., Gonzalez-Moro J.M.R., Paredero J.M., Pérez B., Graziani D., Gutiérrez M., Rodríguez J.M. (2021). Manejo clínico de la EPOC en situación de vida real. Análisis a partir de big data. Arch. Bronconeumol..

[7] Kosteli M.-C., Heneghan N.R., Roskell C., Williams S., Adab P., Dickens A.P., Enocson A., Fitzmaurice D.A., Jolly K., Jordan R. (2017). Barriers and enablers of physical activity engagement for patients with COPD in primary care. Int. J. Chronic Obstr. Pulm. Dis..

[8] Stellefson M.L., Shuster J.J., Chaney B.H., Paige S.R., Alber J.M., Chaney J.D., Sriram P.S. (2017). Web-based Health Information Seeking and eHealth Literacy among Patients Living with Chronic Obstructive Pulmonary Disease (COPD). Health Commun..

[9] Sadeghi S., Brooks D., Stagg-Peterson S., Goldstein R. (2013). Growing Awareness of the Importance of Health Literacy in Individuals with COPD. COPD: J. Chronic Obstr. Pulm. Dis..

[10] Hernandez P., Balter M., Bourbeau J., Hodder R. (2009). Living with chronic obstructive pulmonary disease: A survey of patients’ knowledge and attitudes. Respir. Med..

[11] Leung J.M., Niikura M., Yang C.W.T., Sin D.D. (2020). COVID-19 and COPD. Eur. Respir. J..

[12] Rutkowski S. (2021). Management Challenges in Chronic Obstructive Pulmonary Disease in the COVID-19 Pandemic: Telehealth and Virtual Reality. J. Clin. Med..

[13] Guerra-Paiva S., Dias F., Costaa D., Santos V., Santos C. (2021). DPO2 Project: Telehealth to enhance the social role of physical activity in people living with COPD. Proc. Comput. Sci..

[14] Jansen F., Van Uden-Kraan C.F., Van Zwieten V., Witte B.I., Leeuw I.M.V.-D. (2014). Cancer survivors’ perceived need for supportive care and their attitude towards self-management and eHealth. Support. Care Cancer.

[15] Murray E. (2012). Web-Based Interventions for Behavior Change and Self-Management: Potential, Pitfalls, and Progress. Medicine 2.0.

[16] Slev V.N., Mistiaen P., Pasman H.R.W., Leeuw I.M.V.-D., van Uden-Kraan C.F., Francke A.L. (2016). Effects of eHealth for patients and informal caregivers confronted with cancer: A meta-review. Int. J. Med. Inform..

[17] Gaveikaite V., Grundstrom C., Winter S., Chouvarda I., Maglaveras N., Priori R. (2019). A systematic map and in-depth review of European telehealth interventions efficacy for chronic obstructive pulmonary disease. Respir. Med..

[18] El-Gayar O., Timsina P., Nawar N., Eid W. (2013). A systematic review of IT for diabetes self-management: Are we there yet?. Int. J. Med. Inform..

[19] Bonnevie T., Smondack P., Elkins M., Gouel B., Medrinal C., Combret Y., Muir J.-F., Cuvelier A., Prieur G., Gravier F.-E. (2020). Advanced telehealth technology improves home-based exercise therapy for people with stable chronic obstructive pulmonary disease: A systematic review. J. Physiother..

[20] Hall A.K., Stellefson M., Bernhardt J. (2012). Healthy Aging 2.0: The Potential of New Media and Technology. Prev. Chronic Dis..

[21] Stellefson M., Chaney B., Barry A., Chavarria E., Tennant B., Walsh-Childers K., Sriram P., Zagora J. (2013). Web 2.0 Chronic Disease Self-Management for Older Adults: A Systematic Review. J. Med. Internet Res..

[22] Donner C.F., Raskin J., ZuWallack R., Nici L., Ambrosino N., Balbi B., Blackstock F., Casaburi R., Dreher M., Effing T. (2018). Incorporating telemedicine into the integrated care of the COPD patient a summary of an inter-disciplinary workshop held in Stresa, Italy, 7–8 September 2017. Respir. Med..

[23] Polisena J., Tran K., Cimon K., Hutton B., McGill S., Palmer K., Scott R.E. (2010). Home telehealth for chronic obstructive pulmonary disease: A systematic review and meta-analysis. J. Telemed. Telecare.

[24] Udsen F.W., Hejlesen O., Ehlers L.H. (2014). A systematic review of the cost and cost-effectiveness of telehealth for patients suffering from chronic obstructive pulmonary disease. J. Telemed. Telecare.

[25] Liu F., Jiang Y., Xu G., Ding Z. (2020). Effectiveness of Telemedicine Intervention for Chronic Obstructive Pulmonary Disease in China: A Systematic Review and Meta-Analysis. Telemed. e-Health.

[26] Alwashmi M., Hawboldt J., Davis E., Marra C., Gamble J.-M., Abu Ashour W. (2016). The Effect of Smartphone Interventions on Patients with Chronic Obstructive Pulmonary Disease Exacerbations: A Systematic Review and Meta-Analysis. JMIR mHealth uHealth.

[27] Donner C., ZuWallack R., Nici L. (2021). The Role of Telemedicine in Extending and Enhancing Medical Management of the Patient with Chronic Obstructive Pulmonary Disease. Medicina.

[28] Jang S., Kim Y., Cho W.-K. (2021). A Systematic Review and Meta-Analysis of Telemonitoring Interventions on Severe COPD Exacerbations. Int. J. Environ. Res. Public Health.

[29] Snoswell C.L., Stringer H., Taylor M.L., Caffery L.J., Smith A.C. (2021). An overview of the effect of telehealth on mortality: A systematic review of meta-analyses. J. Telemed. Telecare.

[30] Metting E., Dassen L., Aardoom J., Versluis A., Chavannes N. (2021). Effectiveness of Telemonitoring for Respiratory and Systemic Symptoms of Asthma and COPD: A Narrative Review. Life.

[31] Janjua S., Carter D., Threapleton C.J., Prigmore S., Disler R.T. (2021). Telehealth interventions: Remote monitoring and consultations for people with chronic obstructive pulmonary disease (COPD). Cochrane Database Syst. Rev..

[32] Shaw G., Whelan M.E., Armitage L.C., Roberts N., Farmer A.J. (2020). Are COPD self-management mobile applica-tions effective? A systematic review and meta-analysis. NPJ Prim. Care Respir. Med..

[33] Peters G.M., Kooij L., Lenferink A., van Harten W.H., Doggen C.J.M. (2021). The Effect of Telehealth on Hospital Services Use: Systematic Review and Meta-analysis. J. Med. Internet Res..

[34] Deng N., Gu T., Zhao Q., Zhang X., Zhao F., He H. (2018). Effects of telephone support on exercise capacity and quality of life in patients with chronic obstructive pulmonary disease: A meta-analysis. Psychol. Health Med..

[35] Voncken-Brewster V., Tange H., de Vries H., Nagykaldi Z., Winkens B., van der Weijden T. (2015). A randomized controlled trial evaluating the effectiveness of a web-based, computer-tailored self-management intervention for people with or at risk for COPD. Int. J. Chronic Obstr. Pulm. Dis..

[36] Samoocha D., Bruinvels D.J., Elbers N.A., Anema J.R., van der Beek A.J. (2010). Effectiveness of web-based inter-ventions on patient empowerment: A systematic review and meta-analysis. J. Med. Internet Res..

[37] Pulman A., Lepping P., Paravastu S.C.V., Turner J., Billings P., Minchom P. (2010). A patient centred framework for improving LTC quality of life through Web 2.0 technology. Health Inform. J..

[38] Murray E., Burns J., Tai S.S., Lai R., Nazareth I. (2005). Interactive Health Communication Applications for people with chronic disease. Cochrane Database Syst. Rev..

[39] Bennett G.G., Glasgow R.E. (2009). The delivery of public health interventions via the internet: Actualizing their po-tential. Annu. Rev. Public Health.

[40] Selzler A.M., Wald J., Sedeno M., Jourdain T., Janaudis-Ferreira T., Goldstein R., Bourbeau J., Stickland M.K. (2018). Telehealth pulmonary rehabilitation: A review of the literature and an example of a nationwide initiative to im-prove the accessibility of pulmonary rehabilitation. Chron. Respir. Dis..

[41] Ambrosino N., Vagheggini G., Mazzoleni S., Vitacca M. (2016). Telemedicine in chronic obstructive pulmonary disease. Breathe.

[42] Page M.J., McKenzie J.E., Bossuyt P.M., Boutron I., Hoffmann T.C., Mulrow C.D., Shamseer L., Tetzlaff J.M., Akl E.A., Brennan S.E. (2021). The PRISMA 2020 statement: An updated guideline for reporting systematic reviews. BMJ.

[43] Higgins J.P.T., Altman D.G., Gøtzsche P.C., Jüni P., Moher D., Oxman A.D., Savović J., Schulz K.F., Weeks L., Sterne J.A.C. (2011). The Cochrane Collaboration’s tool for assessing risk of bias in randomised trials. BMJ.

[44] Center for Reviews and Dissemination (2009). Systematic Reviews—CRD’s Guidelines for Undertaking Reviews in Healthcare.

[45] Downs S.H., Black N. (1998). The feasibility of creating a checklist for the assessment of the methodological quality both of randomised and non-randomised studies of health care interventions. J. Epidemiol. Community Health.

[46] Deeks J., Dinnes J., D’Amico R., Sowden A.J., Sakarovitch C., Song F., Petticrew M., Altman D.G. (2003). Evaluating non-randomised intervention studies. Health Technol. Assess..

[47] Saunders L.D., Soomro G.M., Buckingham J., Jamtvedt G., Raina P. (2003). Assessing the Methodological Quality of Nonrandomized Intervention Studies. West. J. Nurs. Res..

[48] Higgins J., Thomas J., Chandler J., Cumpston M., Li T., Page M., Welch V. (2019). Cochrane Handbook for Systematic Reviews of Interventions Version 6.0 (Updated July 2019). Cochrane. www.training.cochrane.org/handbook.

[49] Holroyd-Leduc J.M., Helewa A., Walker J.M. (2002). Critical Evaluation of Research in Physical Rehabilitation: Towards Evidence-Based Practice. Philadelphia: WB Saunders Company, 2000. Evid. Based Med..

[50] Newham J.J., Presseau J., Heslop-Marshall K., Russell S., Ogunbayo O.J., Netts P., Hanratty B., Kaner E. (2017). Features of self-management interventions for people with COPD associated with improved health-related quality of life and reduced emergency department visits: A systematic review and meta-analysis. Int. J. Chronic Obstr. Pulm. Dis..

[51] Blakemore A., Dickens C., Guthrie E., Bower P., Kontopantelis E., Afzal C., Coventry P. (2014). Depression and anxiety predict health-related quality of life in chronic obstructive pulmonary disease: Systematic review and meta-analysis. Int. J. Chronic Obstr. Pulm. Dis..

[52] Cannon D., Buys N., Sriram K.B., Sharma S., Morris N., Sun J. (2016). The effects of chronic obstructive pulmonary disease self-management interventions on improvement of quality of life in COPD patients: A meta-analysis. Respir. Med..

[53] Chaplin E., Hewitt S., Apps L., Bankart J., Pulikottil-Jacob R., Boyce S., Morgan M., Williams J., Singh S. (2017). Interactive web-based pulmonary rehabilitation programme: A randomised controlled feasibility trial. BMJ Open.

[54] Wang L., He L., Tao Y., Sun L., Zheng H., Zheng Y., Shen Y., Liu S., Zhao Y., Wang Y. (2017). Evaluating a Web-Based Coaching Program Using Electronic Health Records for Patients with Chronic Obstructive Pulmonary Disease in China: Randomized Controlled Trial. J. Med. Internet Res..

[55] Nguyen H.Q., Donesky D., Reinke L.F., Wolpin S., Chyall L., Benditt J.O., Paul S.M., Carrieri-Kohlman V. (2012). Internet-Based Dyspnea Self-Management Support for Patients with Chronic Obstructive Pulmonary Disease. J. Pain Symptom Manag..

[56] Moy M.L., Collins R., Martinez C.H., Kadri R., Roman P., Holleman R.G., Kim H.M., Nguyen H.Q., Cohen M.D., Goodrich D. (2015). An Internet-Mediated Pedometer-Based Program Improves Health-Related Quality-of-Life Domains and Daily Step Counts in COPD. Chest.

[57] Moy M.L., Martinez C.H., Kadri R., Roman P., Holleman R.G., Kim H.M., Nguyen H.Q., Cohen M.D., Goodrich D.E., Giardino N.D. (2016). Long-Term Effects of an Internet-Mediated Pedometer-Based Walking Program for Chronic Obstructive Pulmonary Disease: Randomized Controlled Trial. J. Med. Internet Res..

[58] Wan E.S., Kantorowski A., Homsy D., Teylan M., Kadri R., Richardson C.R., Gagnon D.R., Garshick E., Moy M.L. (2017). Promoting physical activity in COPD: Insights from a randomized trial of a web-based intervention and pedometer use. Respir. Med..

[59] Wan E.S., Kantorowski A., Polak M., Kadri R., Richardson C.R., Gagnon D.R., Garshick E., Moy M.L. (2020). Long-term effects of web-based pedometer-mediated intervention on COPD exacerbations. Respir. Med..

[60] Bourne S., Devos R., North M., Chauhan A., Green B., Brown T., Cornelius V., Wilkinson T. (2017). Online versus face-to-face pulmonary rehabilitation for patients with chronic obstructive pulmonary disease: Randomised controlled trial. BMJ Open.

[61] Jiménez-Reguera B., López E.M., Fitch S., Juarros-Monteagudo L., Sánchez-Cortés M., Rodríguez-Hermosa J.L., Calle-Rubio M., Hernández-Criado M.T., López-Martín M., Angulo S. (2020). Development and Preliminary Evaluation of the Effects of an mHealth Web-Based Platform (HappyAir) on Adherence to a Maintenance Program After Pulmonary Rehabilitation in Patients with Chronic Obstructive Pulmonary Disease: Randomized Controlled Trial (Preprint). JMIR mHealth uHealth.

[62] Barak A., Klein B., Proudfoot J.G. (2009). Defining Internet-Supported Therapeutic Interventions. Ann. Behav. Med..

[63] Kuijpers W., Groen W.G., Aaronson N.K., Van Harten W.H. (2013). A Systematic Review of Web-Based Interventions for Patient Empowerment and Physical Activity in Chronic Diseases: Relevance for Cancer Survivors. J. Med. Internet Res..

[64] Gregersen T.L., Green A., Frausing E., Ringbæk T., Brøndum E., Ulrik C.S. (2016). Do telemedical interventions im-prove quality of life in patients with COPD? A systematic review. Int. J. COPD.

[65] Janjua S., Banchoff E., Threapleton C.J.D., Prigmore S., Fletcher J., Disler R.T. (2021). Digital interventions for the management of chronic obstructive pulmonary disease. Cochrane Database Syst. Rev..

[66] Gorst S.L., Armitage C.J., Brownsell S., Hawley M.S. (2014). Home Telehealth Uptake and Continued Use Among Heart Failure and Chronic Obstructive Pulmonary Disease Patients: A Systematic Review. Ann. Behav. Med..

[67] Sul A.R., Lyu D.-H., Park D.-A. (2018). Effectiveness of telemonitoring versus usual care for chronic obstructive pulmonary disease: A systematic review and meta-analysis. J. Telemed. Telecare.

[68] Yohannes A.M. (2012). Telehealthcare management for patients with chronic obstructive pulmonary disease. Expert Rev. Respir. Med..

[69] Kelders S.M., Kok R., Ossebaard H.C., Van Gemert-Pijnen J.E. (2012). Persuasive System Design Does Matter: A Systematic Review of Adherence to Web-based Interventions. J. Med. Internet Res..

[70] Sobnath D.D., Philip N., Kayyali R., Nabhani-Gebara S., Pierscionek B., Vaes A.W., A Spruit M., Kaimakamis E., Mehring M., Ryan D. (2017). Features of a Mobile Support App for Patients with Chronic Obstructive Pulmonary Disease: Literature Review and Current Applications. JMIR mHealth uHealth.

[71] Mcdowell J.E., McClean S., FitzGibbon F., Tate S. (2015). A randomised clinical trial of the effectiveness of home-based health care with telemonitoring in patients with COPD. J. Telemed. Telecare.

[72] Fridriksdottir N., Gunnarsdottir S., Zoëga S., Ingadottir B., Hafsteinsdottir E.J.G. (2018). Effects of web-based inter-ventions on cancer patients’ symptoms: Review of randomized trials. Support. Care Cancer.

[73] Triberti S., Savioni L., Sebri V., Pravettoni G. (2019). eHealth for improving quality of life in breast cancer patients: A systematic review. Cancer Treat. Rev..

[74] Brunton L., Bower P., Sanders C. (2015). The Contradictions of Telehealth User Experience in Chronic Obstructive Pulmonary Disease (COPD): A Qualitative Meta-Synthesis. PLoS ONE.

[75] Ruland C.M., Maffei R.M., Børøsund E., Krahn A., Andersen T., Grimsbø G.H. (2013). Evaluation of different features of an eHealth application for personalized illness management support: Cancer patients’ use and appraisal of usefulness. Int. J. Med. Inform..

[76] McCabe C., McCann M., Brady A.M. (2017). Computer and mobile technology interventions for self-management in chronic obstructive pulmonary disease. Cochrane Database Syst. Rev..

[77] Lundell S., Holmner Å., Rehn B., Nyberg A., Wadell K. (2015). Telehealthcare in COPD: A systematic review and meta-analysis on physical outcomes and dyspnea. Respir. Med..

[78] Eysenbach G., Kummervold P.E., Ritterband L. (2005). “Is Cybermedicine Killing You?”—The Story of a Cochrane Disaster. J. Med. Internet Res..

[79] Welling J.B.A., Hartman J., Hacken N.H.T., Klooster K., Slebos D.-J. (2015). The minimal important difference for the St George’s Respiratory Questionnaire in patients with severe COPD. Eur. Respir. J..

[80] Baumeister R.F., Vohs K.D., Nathan DeWall C., Zhang L. (2007). How Emotion Shapes Behavior: Feedback, Anticipation, and Reflection, Rather Than Direct Causation. Pers. Soc. Psychol. Rev..

[81] De La Torre-Díez I., López-Coronado M., Vaca C., Aguado J.S., de Castro C. (2015). Cost-utility and cost-effectiveness studies of telemedicine, electronic, and mobile health systems in the literature: A systematic review. Telemed. e-Health.

[82] Barbosa M.T., Sousa C.S., Morais-Almeida M., Simões M.J., Mendes P. (2020). Telemedicine in COPD: An Overview by Topics. COPD J. Chronic Obstr. Pulm. Dis..

[83] Kamei T., Yamamoto Y., Kajii F., Nakayama Y., Kawakami C. (2012). Systematic review and meta-analysis of studies involving telehome monitoring-based telenursing for patients with chronic obstructive pulmonary disease. Jpn. J. Nurs. Sci..

[84] Cruz J., Brooks D., Marques A. (2014). Home telemonitoring effectiveness in COPD: A systematic review. Int. J. Clin. Pract..

[85] Hong Y., Lee S.H. (2019). Effectiveness of tele-monitoring by patient severity and intervention type in chronic obstruc-tive pulmonary disease patients: A systematic review and meta-analysis. Int. J. Nurs. Stud..

[86] Wantland D.J., Portillo C.J., Holzemer W.L., Slaughter R., McGhee E.M. (2004). The Effectiveness of Web-Based vs. Non-Web-Based Interventions: A Meta-Analysis of Behavioral Change Outcomes. J. Med. Internet Res..

[87] Eysenbach G. (2005). The law of attrition. J. Med. Internet Res..

[88] Bennett A.V., Jensen R.E., Basch E. (2012). Electronic patient-reported outcome systems in oncology clinical practice. CA A Cancer J. Clin..

